# From E. coli UTI to Spots on the Thigh: A Rare Cause of Erythema Multiforme

**DOI:** 10.7759/cureus.29408

**Published:** 2022-09-21

**Authors:** Elizabeth Sain, Nabeel Ghani, Estelle Vincent, Anuja Sabapathy, Susheel Muralidharan

**Affiliations:** 1 Pediatrics, Lake Erie College of Osteopathic Medicine, Erie, USA; 2 Family Medicine, Allegheny Health Network (AHN) St. Vincent Hospital, Erie, USA; 3 Pediatrics, Allegheny Health Network (AHN) St. Vincent Hospital, Erie, USA

**Keywords:** e. coli, pediatrics, infection, rash, erythema multiforme, uti

## Abstract

Erythema multiforme has largely been associated with microbes such as herpes simplex virus and *Mycoplasma pneumoniae*, drugs, and autoimmune conditions. Here, we report an unusual presentation of erythema multiforme secondary to* Escherichia coli *(*E. coli*) UTI in a pediatric patient. A literature search was conducted and a similar case describing the association between erythema multiforme and hematuria secondary to an *E. coli* UTI in an adult male was identified. However, no case describing *E. coli* manifesting in erythema multiforme has been described in the pediatric patient population previously.

## Introduction

Erythema multiforme (EM) occurs due to an immune reaction most commonly by microbes including herpes simplex virus 1 and 2, and more commonly in the pediatric patient population by *Mycoplasma pneumoniae* [1,2]. Less commonly, drug-immune mediated reaction to classes including sulfonamides, penicillins, barbiturates, non-steroidal anti-inflammatory drugs, statins, and anti-epileptics [1,2]. EM can also occur secondary to vaccine administration, but the incidence is low and debated [1,2]. EM has also been associated with other immune-related conditions such as inflammatory bowel disease and malignancies including leukemia, lymphoma, and gastric adenocarcinoma [2]. This case report seeks to highlight a novel association in a pediatric patient between microbial infection and development of EM [3].

A literature search was conducted and a similar case describing the association between EM and hematuria secondary to an *Escherichia coli *(*E. coli*) UTI in a 66-year-old male was identified [3]. However, no case describing *E. coli* manifesting in EM has been described in the pediatric patient population.

## Case presentation

**Figure 1:**
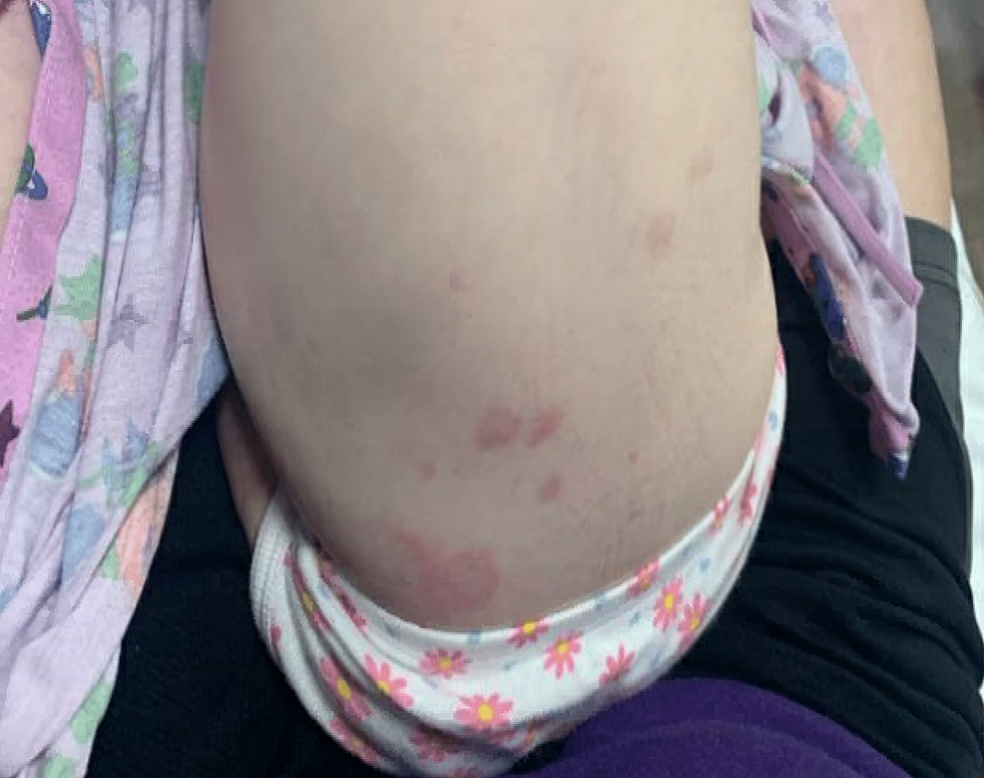
Erythema multiforme rash on patient's back.

**Figure 2:**
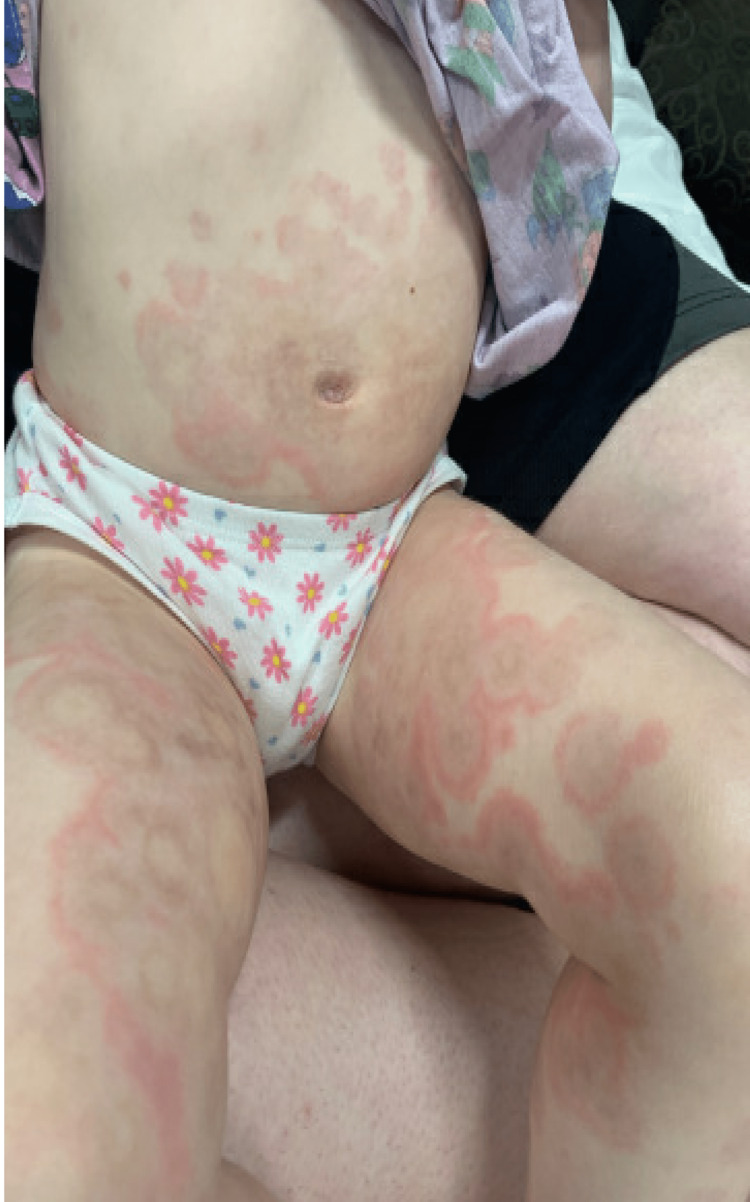
Erythema multiforme rash on patient's abdomen and thighs.

**Figure 3:**
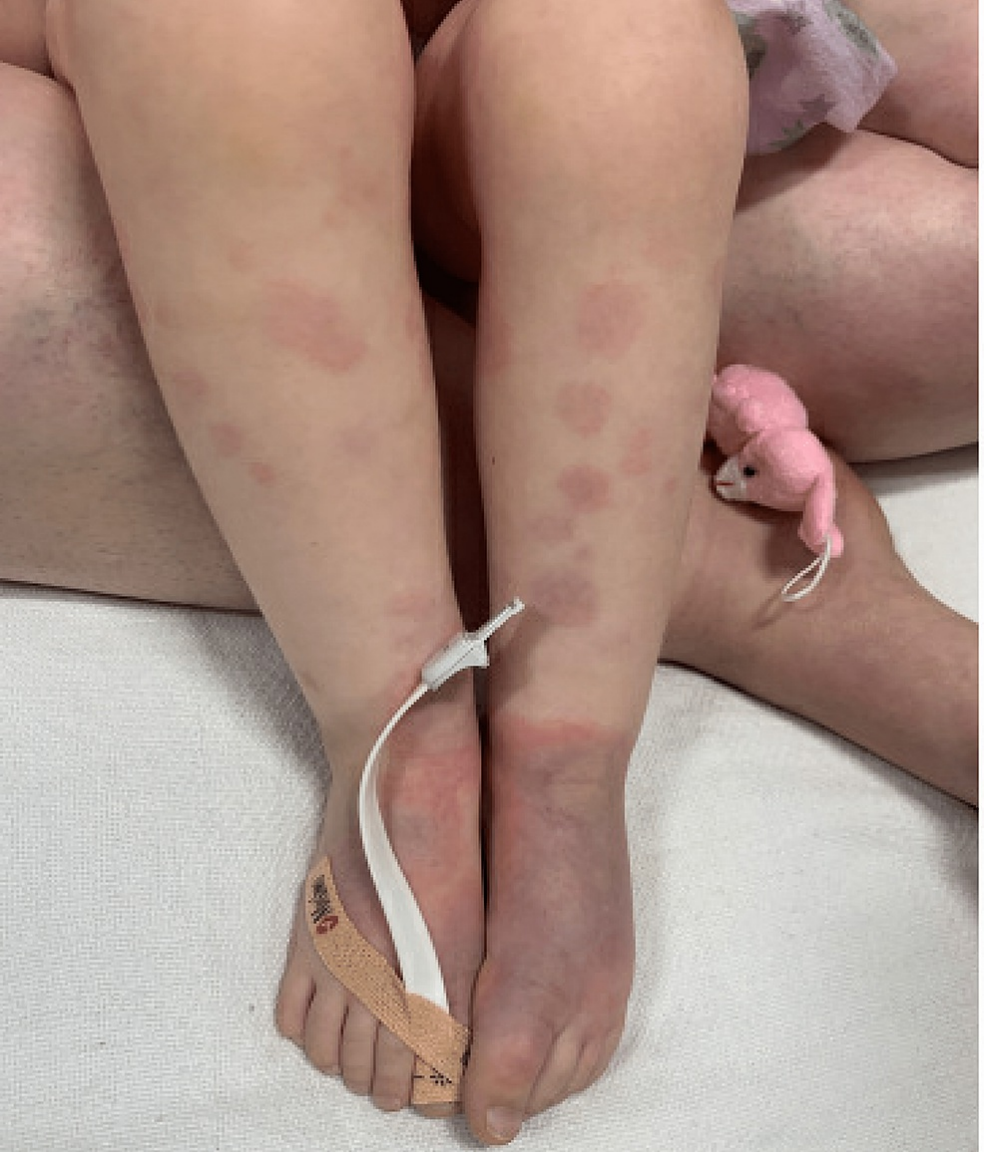
Erythema multiforme rash on patient's legs.

A two-year-old girl with no significant past medical history presented to the emergency department with chief complaints of fever and an itchy rash. Two days prior to presentation, she developed a rash on her thighs bilaterally. The patient was initially evaluated by her primary care physician, who diagnosed the patient with EM. She was treated with ibuprofen, topical steroids, and an antihistamine. Over the course of the next 24 h, the rash spread to most of the patient’s body including her neck, forehead, and jawline. The patient presented to the emergency department the next day with a fever of 40°C (normal range: 36.4-37.2°C), chills, and multiple pruritic skin lesions over the back, abdomen, legs, and hands bilaterally. The skin lesions were described as patches with a dark central spot surrounded by a pale pink ring and a darker erythematous border (Figures 1-3). There was no history of cough, rhinorrhea, nausea, vomiting, or diarrhea. There was no history of cold sores or medication use prior to illness and no history of sick contacts. The patient’s immunizations were up to date. The physical examination was within normal limits except for the aforementioned rash and mild dehydration. There was no involvement of mucous membranes and no abnormal breath sounds.

The patient was admitted to the inpatient pediatric wards. Complete blood count (CBC) showed neutrophilia of 76% (normal range: 21-67%) with a normal white blood cell count of 5,900 per microliter (normal range: 6,300-14,800 per microliter) and normal platelet count of 230,000 per microliter (normal: 200,000- 450,000 per microliter). C-reactive protein (CRP) was elevated at 25.5 mg/L (normal range: 0.8-1 mg/L). A clean catch urine sample was obtained and urinalysis was significant for 2+ blood (normal: negative), trace ketones (normal: negative), trace leukocyte esterase (normal: negative), and 5-10 RBCs/HPF (normal: <2 RBCs/HPF). A chest x-ray was obtained which was negative for consolidative processes.

The patient was treated with acetaminophen and IV fluids for her intermittent fever and poor oral intake. Urine culture returned positive for 10,000-49,000 CFU/mL *E. coli*. Renal ultrasound showed bilateral normal kidneys, ureters, and bladder. The blood culture resulted in no growth.

She was treated with ceftriaxone for her *E. coli* urinary tract infection (UTI). The fluids were discontinued as the patient’s oral liquid intake improved. Prior to discharge, the patient remained afebrile for 24 h, with a significantly improved rash that decreased in size and erythema. Her CRP decreased to 4.2 mg/L (normal range: 0.8-1 mg/L). The patient was discharged home on oral cefixime and a follow-up appointment with her pediatrician was set up.

## Discussion

EM is an autoimmune condition resulting in targetoid, bull's eye-like, lesions that affect the skin [1,2,4]. It is separated into two subgroups - erythema major, when the rash includes mucosal involvement, and erythema minor, when the rash spares the mucosa [2,4]. Historically EM was thought to share the same pathology with other immune-related skin conditions such as Stevens-Johnson syndrome (SJS) and toxic epidermal necrosis (TEN) [1]. Although the lesion formation process is similar, EM typically has minimal epidermal detachment compared to SJS and TEN [1].

Clinically, SJS and TEN manifest as non-specific symptoms of fever, malaise, and upper respiratory tract symptoms in the beginning. Over the next few days, a blistering rash and erosions appear on the body. Usually, the inflammation will form into flaccid bullae and will be painful. There will also be a Nikolsky-positive test resulting from the shedding in the epidermis. Further, mucosal involvement is a very common finding. Given the lack of these findings, SJS and TEN were excluded from the differential diagnosis for this patient.

The histopathology of the targetoid lesions in EM is due to epithelial cell damage via cell-mediated immunity. It begins with the influx of macrophages and T cells which release cytokines resulting in inflammation and damage. If the process is due to drug hypersensitivity, it features necrosis of keratinocytes. The cell-mediated component explains why EM often is associated with infectious agents, both viral and bacterial [5]. The lack of any medication usage prior to her diagnosis of EM excludes any drug-induced causes.

EM is largely a clinical diagnosis assessed through visualization of the targetoid lesion. Laboratory evaluation can reveal leukocytosis, neutropenias, and mild anemia. Treatment for EM involves treating the underlying cause such as *M. pneumoniae* with azithromycin. Systemic corticosteroids and intravenous immunoglobulins have been used without demonstrating their effectiveness [5].

We report a novel case of EM that was not caused by common infectious or pharmaceutical etiologies. Having not found any source of infection or causal factor besides a UTI, it was decided to treat the infection suspecting it to be the cause of the rash. With treatment of the UTI, the rash resolved in the following days. In this case, a direct relationship between treatment of the UTI and the resolution of the rash was shown to be linked.

## Conclusions

The patient’s intermittent high fevers, microscopic hematuria, and elevated CRP were indicative of an infectious, inflammatory response. The infectious etiology was determined to be an *E. coli *UTI. With the rash not having any of the findings similar to SJS or TEN, the patient having no history of pneumonia, blisters, or inciting drug usage, and with the resolution of the rash with the treatment of the UTI, it was concluded that the EM was associated with the *E. coli *UTI. With no other case reports found that also showed this specific association, further research and investigation will be needed in order to demonstrate a causal relationship.
